# Risk of precancerous cervical lesions in women using a hormone-containing intrauterine device and other contraceptives: a register-based cohort study from Denmark

**DOI:** 10.1093/humrep/deab066

**Published:** 2021-05-11

**Authors:** Malene Skorstengaard, Elsebeth Lynge, George Napolitano, Jan Blaakær, Pinar Bor

**Affiliations:** 1 Department of Public Health, University of Copenhagen, Copenhagen, Denmark; 2 Centre for Epidemiological Research, Nykøbing Falster Hospital, University of Copenhagen, Copenhagen, Denmark; 3 Department of Obstetrics and Gynecology, Odense University Hospital, Odense, Denmark; 4 Department of Clinical Research, University of Southern Denmark, Odense, Denmark; 5 Department of Obstetrics and Gynecology, Randers Regional Hospital, Randers, Denmark; 6 Department of Clinical Medicine, Aarhus University, Aarhus, Denmark

**Keywords:** cervical intraepithelial neoplasia / precancerous cervical lesions / hormone-containing intrauterine device / copper intrauterine device / IUD / oral contraceptives / human papillomavirus / contraceptives

## Abstract

**STUDY QUESTION:**

Is the risk of high-grade precancerous cervical lesions and/or is the risk of lesion progression increased in users of a hormone-containing intrauterine device (HIUD) compared with users of other contraceptive methods.

**SUMMARY ANSWER:**

Women starting use of HIUD had the same subsequent risk of cervical intraepithelial neoplasia 3+ (CIN3+) as copper IUD (CIUD) users, and both groups tended to have lower risks than oral contraceptives (OC) users.

**WHAT IS KNOWN ALREADY:**

HIUDs may cause inflammatory and immunosuppressive changes that may potentially affect the risk of persistent human papillomavirus infection and precancerous cervical lesions.

**STUDY DESIGN, SIZE, DURATION:**

A Danish population-based cohort study was conducted using register data from 2008 to 2011 on 26–50-year-old users of HIUD (n = 60 551), CIUD (n = 30 303), or OC (n = 165 627).

**PARTICIPANTS/MATERIALS, SETTING, METHODS:**

Within each user group, women were divided into two groups; normal cytology or abnormal diagnosis before start of contraceptive use (baseline). Follow-up histology and cytology diagnoses were registered during the 5 years after baseline. Adjusted relative risks (aRR) and 95% CI were calculated for precancerous cervical lesions in HIUD users compared with CIUD and OC users.

**MAIN RESULTS AND THE ROLE OF CHANCE:**

Women with normal cytology at baseline: at follow-up HIUD users had the same risk of CIN3 or higher (3+) as CIUD users; aRR 1.08 (95% CI 0.94–1.22). For the HIUD and CIUD groups compared with OCs, the risks of CIN3+ were lower: aRR 0.63 (95% CI 0.57–0.69) and aRR 0.58 (95% CI 0.52–0.65), respectively. The same was observed for CIN2 risks: aRR 0.86 (95% CI 0.76–0.96) and aRR 0.68 (95% CI 0.58–0.79) for HIUD and CIUD groups, respectively. Women with abnormal diagnosis at baseline: a lower progression risk, except for CIN2+ at baseline, was observed in HIUD users compared with OC users. Similar progression risks were found in HIUD and CIUD users. There were no differences between the three contraceptive groups in persistence or regression of present lesions.

**LIMITATIONS, REASONS FOR CAUTION:**

We adjusted for age, education, and region of residence as a proxy for socio-economic factors. Data on smoking and sexual behavior were not available thus we cannot exclude some differences between the three user groups.

**WIDER IMPLICATIONS OF THE FINDINGS:**

These findings suggest that women may safely use HIUDs.

**STUDY FUNDING/COMPETING INTEREST(S):**

A.P. Møller Foundation for the Advancement of Medical Science, Else and Mogens Wedell-Wedellborgs Fund, Direktør Emil C. Hertz og Hustru Inger Hertz Fund, and the Fund for Development of Evidence Based Medicine in Private Specialized Practices. EL is principle investigator for a study with HPV-test-kits provided by Roche. The other authors have nothing to declare.

**TRIAL REGISTRATION NUMBER:**

N/A.

## Introduction

Infection with high-risk human papillomavirus (HPV) is a necessary but not a sufficient cause of cervical cancer (Walboomers *et al.*, 1999). HPV is a common sexually transmitted infection, and 75% of sexually active women will become infected in life (Tota *et al.*, 2011). In Danish women, the prevalence of HPV is highest at age 20–23 years (46%) and decreasing to the lowest prevalence at age 65+ years (5.7%) (Kjær *et al.*, 2014). Most women will clear the infection, but for some women it will persist and may cause precancerous cervical lesions and cancer (Stanley, 2006). Cervical screening aims to find and treat lesions before they progress to cancer.

A hormone-containing intrauterine device (HIUD) is widely used as a preferred contraceptive method and in treatment of irregular bleeding (Hidalgo *et al.*, 2002). In Denmark, the annual number of HIUD sold increased from 15,000 in 2005 to 62,000 in 2017 (Sundhedsdatastyrelsen). Evidence is sparse and diverse on HPV infections and precancerous cervical lesions in women using HIUD compared with women using other contraceptive methods.

One study found that 1 year after insertion, HIUD-users (n = 152) had more persistent HPV infections (*P* = 0.02) and more new HPV infections (*P* = 0.056) than CIUD users (n = 150) (Lekovich *et al.*, 2015). In another study, the HPV infection rate was the same in IUD users (n = 295) as in users of other contraceptive methods (Gavrić-Lovrec and Takač, 2010), and HIUD (n = 187) use did not affect risk of positive cervical cytology and high-grade lesions (Lessard *et al.*, 2008). In a study concerning effect of HIUD use on properties of the mucosal immunity of the upper reproductive tract, both inflammatory and immunosuppressive changes were observed although it was uncertain how these changes would affect the risk of viral infections (Shanmugasundaram *et al.*, 2016). All studies were based on relatively small numbers.

The aim of the present study was to investigate the risk of abnormal cervical cytology and histology after use of HIUD compared with use of other contraceptive methods. First, we hypothesized that HIUD use increases the risk of developing a precancerous cervical lesion. Second, we hypothesized that a precancerous cervical lesion will progress after insertion of an HIUD, given the fact that the presence of cervical dysplasia is listed as a contraindication for insertion of a HIUD (Pro.medicin).

## Materials and methods

### Setting

In Denmark, women aged 23 years are invited to cervical screening every 3 years until age 50 years, whereafter they are invited every 5 years, and women aged 60–64 years are offered an HPV-checkout-test (Sundhedsstyrelsen, 2012). The screening test is a liquid-based cytology collected by the general practitioner (GP), and if severely or repeatedly abnormal, the woman is referred to an office gynecologist or a hospital out-patient clinic for colposcopy and biopsies. In Denmark, HPV co-testing is not used, and HPV status at time of recruitment is, therefore, not known. An IUD can be inserted by a GP, an office gynecologist, or at a hospital. Oral contraceptives (OC) are prescriptive drugs, and can be bought only at a registered pharmacy. Primary and secondary healthcare is free of charge for all citizens in Denmark.

### Data sources and diagnoses

All Danish citizens have unique identification numbers, allowing linkage between registers. From the Central Population Register we retrieved data on sex, region of residence, date of birth, death, immigration, and emigration (Schmidt *et al.*, 2014). Data on cervical cytology and histology diagnoses were retrieved from the National Pathology Register (Bjerregaard and Larsen, 2011). From the Prescription Register, we had information on prescribed and purchased contraceptives (Wallach Kildemoes *et al.*, 2011). The National Patient Register holds information on hospital contacts including dates and diagnostic/procedure codes (Schmidt *et al.*, 2015). Procedures performed by office gynecologists and GPs were retrieved from the National Health Services Register, where services are registered by reimbursement date (Sahl Andersen *et al.*, 2011). From the Education Register, we retrieved data on highest achieved education before age 32 years.

In Denmark, pathological specimens are coded with topography (T-code) and morphology (M-code) codes (Patobank). Since 2012, the cervical intraepithelial neoplasia (CIN) classification has been used for morphology coding of histology. The conversion table of the Danish Quality Assurance of the Cervical Cancer Screening Program (DKLS, 2013) was used to convert former codes into the CIN codes. We divided histology diagnoses into: normal; CIN1; CIN2; CIN3; and cancer. For cytology, the Bethesda classification was implemented gradually since 2007, and former cytology codes were converted to Bethesda codes (DKLS, 2013). Cytology diagnoses were divided into: negative for intraepithelial lesion or malignancy (NILM); atypical squamous cells of undetermined significance (ASCUS) including also atypical glandular cells (AGC); low-grade squamous intraepithelial lesion (LSIL); and high-grade squamous intraepithelial lesion (HSIL) including also atypical squamous cells cannot exclude HSIL (ASC-H). Moreover, both histology and cytology included ‘unsatisfactory’ samples that could not be analyzed, and ‘other’ samples with codes that could not be translated.

### Study population and outcomes

We conducted a cohort study using Danish national register data including women aged 26–50 years between 1 January 2008 and 31 December 2011, as they could need contraception or bleeding regulation, and had at least one invitation to screening in the study period. We studied three mutually exclusive groups: HIUD users, CIUD users, and OC users (Supplementary Fig. S1). The earliest IUD insertion or OC purchase date in the 4-year inclusion period was used as index date.

#### HIUD group

Women were included if a HIUD was bought at a pharmacy based on a prescription, and the device was inserted at a hospital, by an office gynecologist, or by a GP. We allowed a maximum of 6 months delay from pharmacy purchase to insertion, and an extra 9 days for registration of insertion. Women were excluded if they had an IUD removal code at a hospital, an office gynecologist, or a GP for up to 3 years before the index date. The Mirena^®^ hormone-containing IUD, a levonorgestrel releasing device, was the only approved HIUD in the study period (Danish Medicines Agency; Sundhedsdatastyrelsen). We did not exclude women who might also have used OC at any point during the study period.

#### CIUD group

Women having a CIUD inserted at a hospital, by an office gynecologist, or by a GP. In Denmark, CIUDs can be bought without a prescription. From the hospital codes we could distinguish between insertion of a HIUD or a CIUD. For the office gynecologists and GPs, inserted IUD-type was not coded, and a woman was, therefore, included in the CIUD group, if there was an IUD insertion code without HIUD purchase for up to 6 months before start of study period (1 July 2007) until 9 days after the end of study period (9 January 2012). We excluded women who had an IUD removal code at a hospital, an office gynecologist, or a GP for up to 3 years before index date and women who bought an HIUD up to 3 years before or 5 years after index date. We did not exclude women who might also have used OC at any point during the study period.

#### OC group

Women were included if they bought OC at least once based on a prescription at a pharmacy between 1 January 2008 and 31 December 2011. Women were excluded if they had purchased an IUD or had an IUD insertion/removal at the hospital, by an office gynecologist, or by a GP up to 3 years before or 5 years after they bought the OC.

We distinguished between baseline and follow-up diagnoses. The baseline diagnosis was the most severe cytological or histological diagnosis during the 3 years before the index date. Similarly, the follow-up diagnosis was the most severe diagnosis during the 5 years after the index date, as both IUD types need to be replaced after 5 years (Pro.medicin). Some women were excluded from the analysis. First, to have complete diagnostic information and to ensure equal follow-up time, we excluded women who immigrated, emigrated, died or disappeared during the 3 years before the index date and 5 years after. Second, women without a cytology or histology 3 years before the index date were excluded (Supplementary Fig. S2). Third, women diagnosed with an immunosuppressive disease and/or with a prescription of immunosuppressive medicine in the 3 years before and 5 years after the index date were excluded, because they have an increased risk of cervical dysplasia (Kane *et al.*, 2008; Zard *et al.*, 2014; Dugué *et al.*, 2015) (Supplementary Fig. S1).

To identify the most severe baseline/follow-up diagnosis, we used a pre-defined hierarchy with a histology diagnosis as the most severe in the following order; cancer, CIN3, CIN2, CIN1, normal histology, unsatisfactory histology, other histology, and in the absence of histology with a cytology diagnosis as the most severe in the following order: HSIL, LSIL, ASC-US, NILM, unsatisfactory cytology, and other cytology. Progression was defined as: follow-up diagnosis more severe than baseline diagnosis. *Persistence*: same diagnosis at baseline and follow-up, including normal histology at baseline. *Regression*: baseline diagnosis more severe than follow-up diagnosis. See Supplementary Table SI for a complete description. For region of residence, we used the five Danish regions: Central Denmark Region, North Denmark Region, Region Zealand, Region of Southern Denmark and the Capital Region. Education was classified into five categories: primary and lower secondary, upper secondary, short cycle tertiary, bachelor/master/doctorial or equivalent, and not elsewhere classified or missing.

**Figure 1.**
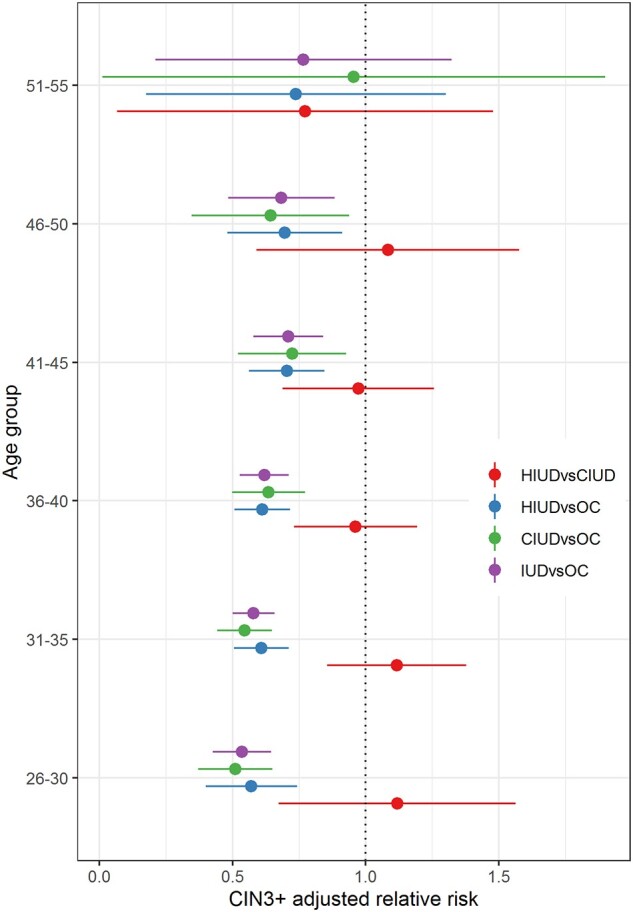
**Adjusted relative risks of cervical intraepithelial neoplasia 3+ stratified by age groups in HIUD, CIUD, and OC users**. CIN3+, cervical intraepithelial neoplasia 3+; HIUD, hormone intrauterine device; CIUD, copper intrauterine device; OC, oral contraceptives.

First, we analyzed women who had normal cytology only during the 3 years preceding their index date. We compared follow-up diagnoses between the three contraceptive user groups. Also, relative risks (RR) of CIN3+ and 95% CIs were calculated, stratified by age (Fig. 1, Supplementary Table SII). Second, we analyzed women with any abnormal cytology/histology during the 3 years preceding their index data. We compared progression, persistence, and regression among the three contraceptive user groups, stratified by the baseline diagnosis.

**Figure 2.**
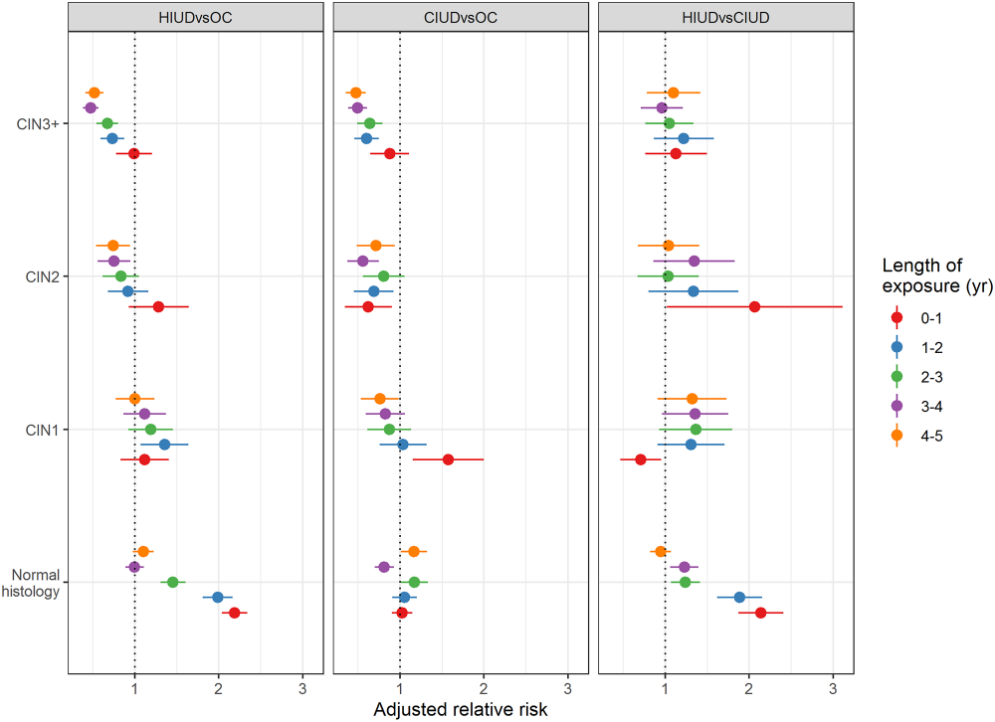
**Adjusted relative risks of histology diagnoses at follow-up in HIUD, CIUD, and OC users with normal cytology at baseline, stratified by length of contraceptive use**.

We calculated cumulative exposure time from index date to the follow-up diagnosis for women in each contraceptive group. In the IUD groups, this was defined as the sum of all exposure time intervals between the dates of an IUD insertion and following removal code (or date of diagnosis, if no removal code was found after an insertion). See Supplementary Data for a more detailed description. For women in the OC group, the exposure time was computed by adding 3 months for each OC prescription purchase between the date of the index event and date of follow-up diagnosis, as a prescription provides three OC packages, each of 1-month duration. Exposure length was divided into; 0–1 year, 1–2 years, 2–3 years, 3–4 years, and 4–5 years (Supplementary Table SIII). The adjusted RR of histology diagnoses in HIUD versus OC, CIUD versus OC, and HIUD versus CIUD by different length of exposure were computed (Fig. 2).

A comparison was made between our results and two previous studies investigating the risk of precancerous cervical lesions associated with use of HIUD, CIUD, and OC (Averbach *et al.*, 2018; Loopik *et al.*, 2020).

### Statistical analysis

For the follow-up diagnosis, as well as for progression, persistence and regression, we computed RR and 95% CI for HIUD versus OC, CIUD versus OC, and HIUD versus CIUD. To account for possible differences between groups (age, socio-economic status, time between diagnoses, length of exposure), the RRs were adjusted for time interval between baseline and follow-up diagnoses, age at follow-up, region of residence at follow-up, education, and length of exposure. Data on race and health behavior were not available.

Crude RRs and 95% CIs were calculated with a multinomial logistic regression model. Adjusted RR (aRR) and 95% CI were calculated with a logistic regression model. Pseudo-anonymized register data were accessed at Statistics Denmark. SAS statistical software version 9.4 (SAS Institute Inc., Cary, NC, USA), together with NLEstimate and NLMeans macros, was used for the analysis. Plots were made in R version 3.6.3 (R Core Team, 2020), using ggplot2 package (Wickham, 2016).

### Ethics

The Danish Data Protection Agency (SUND-2017-52) approved use of data. In Denmark, approval from an Ethics Committee is not required for register-based projects.

## Results

### Study population

We identified 72 125 HIUD users, 39 291 CIUD users, and 236 225 OC users, of whom, 11 574 (16.1%) were excluded in the HIUD group, 8988 (22.9%) in the CIUD group, and 70 598 (29.9%) in the OC group, mainly due to missing baseline diagnosis. The HIUD, CIUD, and OC study groups finally included 60 551, 30 303, and 165 627 women, respectively, Table I.

**Table I:** Characteristics of users of hormonal intrauterine devices, copper intrauterine devices and oral contraceptives aged 26–50 years in Denmark, 2008–2011.

	HIUD	CIUD	OC
Number of women (*n*, %)	72 125	39 291	236 225
Excluded for no baseline diagnosis (*n*, %)	8015 (11.1%)	6874 (17.5%)	57 286 (24.2%)
Excluded for missing follow-up (*n*, %)	3541 (4.9%)	2093 (5.3%)	13 176 (5.6%)
Excluded for no known residence in DK at follow-up (*n*, %)	18 (<0.1%)	21 (0.1%)	136 (0.1%)
Number of included women (*n*, %)	60 551 (83.9%)	30 303 (77.1%)	165 627 (70.1%)
Baseline diagnosis			
- Normal cytology	53 283 (88.0%)	27 222 (89.8%)	146 818 (88.6%)
- Abnormal diagnosis	7268 (12.0%)	3081 (10.2%)	18 809 (11.4%)
Mean age (SD) at index event (years)	38.7 (5.9)	36.0 (6.1)	33.4 (6.6)
- Normal cytology at baseline	38.6 (5.8)	36.1 (6.1)	33.6 (6.6)
- Abnormal diagnosis at baseline	39.4 (6.1)	35.3 (6.4)	32.0 (6.3)
Mean age (SD) at follow-up (years)	41.1 (6.0)	38.3 (6.3)	35.5 (6.8)
- Normal cytology at baseline	41.0 (6.0)	38.5 (6.3)	35.7 (6.8)
- Abnormal diagnosis at baseline	41.4 (6.4)	37.1 (6.6)	33.6 (6.6)
Region of residence at follow-up (*n*, %)			
- Capital Region	18 718 (30.9%)	12 868 (42.5%)	56 087 (33.9%)
- Central Denmark	15 289 (25.2%)	5721 (18.9%)	34 967 (21.1%)
- Northern Denmark	5708 (9.4%)	2496 (8.2%)	16 881 (10.2%)
- Southern Denmark	11 617 (19.2%)	5487 (18.1%)	35 590 (21.5%)
- Zealand	9219 (15.2%)	3731 (12.3%)	22 102 (13.3%)
Highest education level before age 32 years (*n*, %)			
- Primary and lower secondary	6834 (11.3%)	3468 (11.4%)	19 519 (11.8%)
- Upper secondary	25 156 (41.5%)	10 792 (35.6%)	67 489 (40.7%)
- Short cycle tertiary	3265 (5.4%)	1653 (5.5%)	9814 (5.9%)
- Bachelor/Master/Doctorial or equivalent	21 352 (35.3%)	12 761 (42.1%)	63 050 (38.1%)
- Not elsewhere classified or missing	3944 (6.5%)	1629 (5.4%)	5755 (3.5%)
Mean time (SD) to diagnosis at follow-up (years)	3.8 (1.4)	3.8 (1.4)	3.8 (1.4)
- Normal cytology at baseline	3.9 (1.4)	3.9 (1.3)	3.8 (1.3)
- Abnormal diagnosis at baseline	3.1 (1.8)	3.1 (1.8)	3.1 (1.8)
Median time (range) to diagnosis at follow-up (years)	3.3 (8.0)	3.3 (8.0)	3.3 (8.0)
- Normal cytology at baseline	3.4 (8.0)	3.4 (8.0)	3.4 (8.0)
- Abnormal diagnosis at baseline	3.1 (7.9)	3.1 (7.8)	3.1 (8.0)
Length of exposure in years (yr) (*n*, %)			
- 0 < yr ≤ 1	12 283 (20.3%)	6568 (21.7%)	75 706 (45.7%)
- 1 < yr ≤ 2	12 909 (21.3%)	6602 (21.8%)	41 430 (25.0%)
- 2 < yr ≤ 3	15 340 (25.3%)	7231 (23.9%)	26 488 (16.0%)
- 3 < yr ≤ 4	12 903 (21.3%)	6579 (21.7%)	14 244 (8.6%)
- 4 < yr ≤ 5	7116 (11.8%)	3323 (11.0%)	7759 (4.9%)

DK, Denmark; HIUD: hormone intrauterine device, CIUD: copper intrauterine device, OC: oral contraceptives.

The mean age at index date and at follow-up was the lowest (33.4 and 35.5 years, respectively) for women using OC, and the highest (38.7 and 41.1 years, respectively) for women using HIUD; similar results were found in women with normal and abnormal diagnosis at baseline. Mean time to follow-up was fairly similar in the three groups: 1383 days for HIUD, 1380 for CIUD, and 1370 for OC.

Slightly more women using HIUD (25.2%) lived in the Central Denmark Region than those using CIUD (18.9%) or OC (21.1%), while more women using CIUD (42.5%) lived in the Capital Region than those using HIUD (30.9%) or OC (33.9%), *P* < 0.0001 (Chi-squared test for homogeneity). Distribution by education looked similar, but still statistically significantly different (*P* < 0.0001), with more CIUD users achieving a bachelor/higher degree compared with the other groups (Chi-squared test for homogeneity).

### Normal diagnosis at baseline

Of the included women, 53 283 (88%) in the HIUD group, 27 222 (90%) in the CIUD group, and 146 818 (89%) in the OC group had normal cytology at baseline (Table I), and the majority of these women had a normal cytology at follow-up: 84.7%, 88.6%, and 87.0%, respectively.

Normal histology was diagnosed in 8.3% of HIUD users, 5.1% of CIUD users, and in 4.4% of OC users: this left 5.4%, 5.7%, and 7.9%, respectively, with either a histological or cytological abnormality (Table II).

**Table II:** Worst diagnosis (histology and cytology in the absence of histology) at follow-up for women with normal cytology at baseline.

	Groups			Crude relative risks (RR (95%CI))			Adjusted relative risks** (aRR (95%CI))		
Most severe follow-up diagnosis	HIUD N = 53 283	CIUD N = 27 222	OC N = 146 818	HIUD versus OC	CIUD versus OC	HIUD versus CIUD	HIUD versus OC	CIUD versus OC	HIUD versus CIUD
Histology	6193 (11.6)	2298 (8.4)	14 000 (9.5)						
CIN3+	645 (1.2%)	373 (1.4%)	3810 (2.6%)	0.47 (0.43–0.51)	0.53 (0.47–0.58)	0.88 (0.77–1.00)	0.63 (0.57–0.69)	0.58 (0.52–0.65)	1.08 (0.94–1.22)
CIN2	413 (0.8%)	197 (0.7%)	1629 (1.1%)	0.70 (0.62–0.77)	0.65 (0.56–0.75)	1.07 (0.89–1.25)	0.86 (0.75–0.96)	0.68 (0.57–0.78)	1.27 (1.05–1.48)
CIN1	641 (1.2%)	296 (1.1%)	1794 (1.2%)	0.98 (0.90–1.07)	0.89 (0.78–1.00)	1.11 (0.95–1.26)	1.15 (1.03–1.27)	0.95 (0.83–1.07)	1.21 (1.05–1.38)
Normal histology	4408 (8.3%)	1395 (5.1%)	6466 (4.4%)	1.88 (1.81–1.95)	1.16 (1.10–1.23)	1.61 (1.52–1.71)	1.67 (1.60–1.74)	1.14 (1.07–1.21)	1.46 (1.38–1.55)
Undefined^*^ hist	86 (0.2%)	37 (0.1%)	301 (0.2%)	0.79 (0.60–0.98)	0.66 (0.44–0.89)	1.19 (0.73–1.64)	0.69 (0.51–0.87)	0.63 (0.41–0.85)	1.09 (0.67–1.52)
Cytology	47 090 (88.4)	24 924 (91.6)	132 818 (90.4)						
ASCUS+	1189 (2.2%)	673 (2.5%)	4356 (3.0%)	0.75 (0.70–0.80)	0.83 (0.77–0.90)	0.90 (0.82–0.99)	0.77 (0.72–0.83)	0.85 (0.78–0.93)	0.90 (0.82–0.99)
Normal cytology	45 108 (84.7%)	24 130 (88.6%)	127 793 (87.0%)	0.97 (0.97–0.98)	1.02 (1.01–1.02)	0.96 (0.95–0.96)	0.97 (0.97–0.98)	1.02 (1.01–1.02)	0.95 (0.95–0.96)
Undefined^*^ cyt	793 (1.5%)	121 (0.4%)	669 (0.5%)	3.27 (2.93–3.60)	0.98 (0.79–1.16)	3.35 (2.71–3.99)	3.04 (2.69–3.40)	1.11 (0.89–1.34)	2.74 (2.21–3.26)

Data are crude and adjusted RR with 95% CI. N, number.

* Other/unsatisfactory.

** Adjusted for age, region of residence, education level before age 32 years, time to follow-up, and duration of IUD or OC use.

The aRR of normal cytology at follow-up was close to 1 both when comparing HIUD use with OC, aRR = 0.97 (95% CI 0.97–0.98); CIUD use with OC, aRR = 1.02 (95% CI 1.01–1.02); and HIUD with CIUD, aRR = 0.95 (95% CI 0.95–0.96) (Table II). Normal histology was more frequently diagnosed in HIUD users than in CIUD users, aRR = 1.46 (95% CI 1.38–1.55), and OC users, aRR = 1.67 (95% CI 1.60–1.74). In women diagnosed with abnormal histology at follow-up, a lower risk of CIN2 and CIN3+ was observed in CIUD users compared with OC; aRR = 0.68 (95% CI 0.57–0.78) and aRR = 0.58 (95% CI 0.52–0.65), respectively. Also, a lower risk of CIN2, aRR = 0.86 (95% CI 0.75–0.96), and CIN3+, aRR= 0.63 (95% CI 0.57–0.69), was observed in HIUD users compared with OC use. There was no difference in risk of CIN3+ for HIUD compared with CIUD users, aRR = 1.08 (95% CI 0.94–1.22). Higher risk of CIN1 was found in HIUD compared with CIUD: aRR = 1.15 (95% CI 1.03–1.27). There was about a 3-fold increased risk of undefined cytology in HIUD compared with OC and CIUD.

Similar results were found in the age-stratified analysis. The aRR and 95% CIs of CIN3+ were similar in each age group, with a lower risk in HIUD than in OC users, ranging from aRR = 0.54 (95% CI 0.43–0.65), for the age-group 26–30 years, to aRR = 0.70 (95% CI 0.48–0.91), for the age-group 46–50 years. Numbers were small in the age-group 51–55 years. A similar pattern was seen for the comparison between CIUD and OC users (Fig. 1 and Supplementary Table SII).

The overall lower risk of CIN3+ in HIUD and CIUD users than in OC users prevailed when the data were stratified by length of use (Supplementary Table SIII), even with a tendency for the aRRs of CIN3+, CIN2, and CIN1 (HIUD and CIUD versus OC) to decrease with length of use (Fig. 2 and Supplementary Table SIII). On the other hand, the risk of abnormal histology was similar in HIUD users compared with CIUD users in all strata. An excess risk of normal histology for HIUD users compared with both CIUD and OC users was seen in particular in women with only 1–2 years of use (Fig. 2 and Supplementary Table SIII).

### Abnormal diagnosis at baseline

There were 7268 (12%) HIUD users, 3081 (10%) CIUD users, and 18 809 (11%) OC users with abnormal diagnosis at baseline (Table III). The risk of progression was not different in HIUD users compared with OC users for CIN2+ at baseline aRR= 1.16 (95% CI 0.73–1.59) whereas the risk was slightly lower for CIN1 aRR= 0.72 (95% CI 0.51–0.93), normal histology aRR= 0.74 (95% CI 0.60–0.87), and ASCUS+ aRR= 0.83 (95% CI 0.75–0.92). The risk of progression in HIUD users was not significantly different compared with CIUD users for CIN2+ at baseline aRR= 1.44 (95% CI 0.61–2.26), CIN1 aRR= 0.87 (95% CI 0.53–1.22), normal histology aRR= 0.82 (95% CI 0.62–1.03), and ASCUS+ aRR= 0.95 (95% CI 0.82–1.08). The risk of progression of ASCUS+ in CIUD users was lower than in OC users, aRR = 0.88 (95% CI 0.78–0.98) but otherwise the same for CIN2+, CIN1, and normal histology. Neither persistence nor regression were increased in any of the comparisons between contraceptive groups and by diagnosis at baseline (Table III).

**Table III:** Progression, persistence, and regression for women with abnormal diagnosis at baseline.

Baseline diagnosis	Change	Groups			Crude relative risks (RR (95%CI))			Adjusted relative risks** (aRR (95%CI))		
		HIUD n = 7268	CIUD n = 3081	**OC n = 18** **809**	HIUD versus OC	CIUD versus OC	HIUD versus CIUD	HIUD versus OC	CIUD versus OC	HIUD versus CIUD
CIN2+	Progression	42 (3.5)	16 (2.3)	170 (3.1)	1.14 (0.76–1.52)	0.73 (0.36–1.10)	1.57 (0.68–2.46)	1.16 (0.73–1.59)	0.81 (0.39–1.23)	1.44 (0.61–2.26)
	Persistence	74 (6.2)	48 (6.8)	344 (6.3)	1.00 (0.75–1.24)	1.08 (0.77–1.40)	0.92 (0.60–1.25)	0.89 (0.65–1.13)	1.12 (0.78–1.45)	0.79 (0.51–1.08)
	Regression	1048 (88.4)	634 (89.3)	4915 (89.4)	0.99 (0.97–1.01)	1.00 (0.97–1.03)	0.99 (0.96–1.02)	1.00 (0.97–1.03)	1.00 (0.96–1.04)	1.00 (0.95–1.04)
	Undefined^*^	22 (1.9)	12 (1.7)	67 (1.2)	1.52 (0.80–2.25)	1.39 (0.54–2.23)	1.10 (0.33–1.86)	1.36 (0.63–2.09)	1.10 (0.40–1.80)	1.23 (0.34–2.12)
	Total	1186 (16.3)	710 (23.0)	5496 (29.2)						
CIN1	Progression	58 (11.4)	34 (11.9)	260 (14.1)	0.81 (0.60–1.03)	0.85 (0.56–1.13)	0.96 (0.58–1.35)	0.72 (0.51–0.93)	0.82 (0.55–1.08)	0.87 (0.53–1.22)
	Persistence	62 (12.2)	31 (10.8)	208 (11.2)	1.09 (0.80–1.38)	0.96 (0.62–1.31)	1.13 (0.67–1.59)	0.88 (0.60–1.17)	0.86 (0.53–1.19)	1.02 (0.58–1.47)
	Regression	377 (74.4)	217 (75.9)	1363 (73.7)	1.01 (0.95–1.07)	1.03 (0.96–1.10)	0.98 (0.90–1.06)	1.09 (1.00–1.18)	1.08 (0.98–1.18)	1.01 (0.90–1.12)
	Undefined^*^	10 (2.0)	4 (1.4)	18 (1.0)	2.03 (0.47–3.58)	1.44 (0.00–2.98)	1.41 (0.00–3.03)	2.26 (0.14–4.38)	1.39 (0.00–2.98)	1.62 (0.00–3.58)
	Total	507 (7.0)	286 (9.3)	1849 (9.8)						
Normal histology	Progression	228 (6.6)	80 (9.2)	478 (11.6)	0.57 (0.48–0.65)	0.79 (0.62–0.97)	0.71 (0.54–0.89)	0.74 (0.60–0.87)	0.89 (0.68–1.11)	0.82 (0.62–1.03)
	Persistence	3138 (90.6)	776 (89.7)	3568 (86.9)	1.04 (1.03–1.06)	1.03 (1.01–1.06)	1.01 (0.98–1.04)	1.01 (0.99–1.03)	1.02 (0.99–1.04)	1.00 (0.97–1.02)
	Regression	–	–	–	–	–	–	–	–	–
	Undefined^*^	97 (2.8)	9 (1.0)	58 (1.4)	1.98 (1.34–2.62)	0.74 (0.22–1.25)	2.69 (0.86–4.52)	2.10 (1.28–2.92)	0.80 (0.22–1.37)	2.64 (0.82–4.46)
	Total	3463 (47.6)	865 (28.1)	4104 (21.8)						
ASCUS+	Progression	375 (22.5)	249 (26.3)	1561 (27.5)	0.82 (0.74–0.90)	0.96 (0.85–1.07)	0.86 (0.74–0.98)	0.83 (0.75–0.92)	0.88 (0.78–0.98)	0.95 (0.82–1.08)
	Persistence	46 (2.8)	26 (2.7)	156 (2.7)	1.01 (0.68–1.33)	1.00 (0.59–1.41)	1.01 (0.53–1.48)	0.83 (0.52–1.15)	0.97 (0.55–1.39)	0.86 (0.44–1.27)
	Regression	1227 (73.8)	664 (70.2)	3934 (69.3)	1.06 (1.03–1.10)	1.01 (0.97–1.06)	1.05 (1.00–1.10)	1.10 (1.05–1.16)	1.07 (1.01–1.13)	1.03 (0.97–1.10)
	Undefined^*^	15 (0.9)	7 (0.7)	27 (0.5)	1.90 (0.70–3.09)	1.56 (0.27–2.85)	1.22 (0.13–2.31)	1.17 (0.30–2.04)	1.23 (0.13–2.34)	0.95 (0.08–1.82)
	Total	1663 (22.9)	946 (30.7)	5678 (30.2)						
Undefined histology	Undefined	404 (100)	263 (100)	1612 (100)	–	–	–	–	–	–
Undefined cytology	Undefined	45 (100)	11 (100)	70 (100)	–	–	–	–	–	–

* Other/unsatisfactory.

** Adjusted for age, region of residence, education level before 32 years, time to follow-up, and duration of IUD or OC use.

Results of a comparison between our results and two previous studies investigating the risk of precancerous cervical lesions associated with use of HIUD, CIUD, and OC appear in Table IV.

**Table IV:** Associations between IUD use and later CIN3+: summary of literature and own findings.

Comparison group (= unexposed)	Risk group (=exposed)			
	All IUD	HIUD	CIUD	OC
Non-users of contraceptives	RR 1.51 (1.32–1.74) (Loopik *et al.*, 2020)	NA	NA	RR 2.77 (2.56–3.00) (Loopik *et al.*, 2020)
Non-users of IUD	OR 0.98 (0.90–1.07) (Averbach *et al.*, 2018)	OR 1.05 (0.91–1.16) (Averbach *et al.*, 2018)	OR 0.81 (0.64–1.02) (Averbach *et al.*, 2018)	NA
Users of other hormonal contraceptives	OR 0.94 (0.80–1.10) (Averbach *et al.*, 2018)	NA	NA	NR
OC users	RR 0.55 (0.48–0.63)^*^ (Loopik *et al.*, 2020)	aRR 0.63 (0.57–0.69) Present study	aRR 0.58 (0.52–0.65) Present study	NR
CIUD users	–	aRR 1.08 (0.94–1.22) Present study	NR	aRR 1.72 (1.53–1.92)^*^ Present study

Data are RR/odds ratio (OR) and 95% CI. NA, not available; NR, not relevant.

* Calculated as reciprocal of reported values.

## Discussion

### Main findings

We investigated the association between contraceptive use and risk of developing precancerous cervical lesions or experiencing progression of an existing lesion.

In women with normal cytology at the time of initiating contraceptive use, we found that HIUD and CIUD users over the next 5 years had a lower risk of CIN2 and CIN3+ than OC users. Users of HIUD were more likely to have a normal histology or low grade CIN1 diagnosis than women using either CIUD or OC. This was in particular seen in women with only 1–2 years of HIUD use, and may possibly be explained by diagnostic follow-up of irregular bleeding following the HIUD insertion. Among women followed up with cytology only, HIUD and CIUD users had lower risk of abnormalities than OC users.

In women with an existing abnormality at the time of initiating contraceptive use, we found that progression of this abnormality occurred with equal frequency in the three user groups, except for a slight protection against progression of less severe precancerous cervical lesions in HIUD users. A possible explanation for this similarity may be that, in Denmark, women with CIN3+ and women with CIN2 without a pregnancy wish are always treated with a conization to prevent lesion progression. The risk of persistence and regression was equal between the three contraceptive user groups.

### Other studies

Two meta-analyses including mainly case–control studies of patients with cervical cancer found IUD use, HIUD and CIUD combined, as compared with non-IUD use, to be associated with a lower risk of cervical cancer (Castellsagué *et al.*, 2011; Cortessis *et al.*, 2017). As non-IUD users may include both OC users and women not using contraceptives, it is difficult to say whether these findings indicate a true protective effect of IUD use.

In a US case–control study, IUD use compared with non-IUD use did not affect the risk of CIN3+; odds ratio (OR) 0.98 (95% CI 0.90–1.07) and only marginally for CIN2+; OR 1.09 (95% CI 1.03–1.16), and this pattern was the same when the comparison was made with users of other hormonal contraceptives. Compared with the non-IUD users, the OR for CIN3+ for HIUD was 1.05 (95% CI 0.91–1.21) and for CIUD it was 0.81 (95% CI 0.64–1.02), with the slight excess risk for CIN2+ in all IUD-users coming from the HIUD group; OR 1.18 (95% CI 1.08–1.30) (Averbach *et al.*, 2018). Independent of comparison group, this study indicated limited impact of IUD use on the risk of high-grade cervical lesions.

In a large cohort study from the Netherlands, IUD and OC users had an excess risk for CIN3+; RR 1.51 (95% CI 1.32–1.74) and RR 2.77 (95% CI 2.56–3.00), respectively, compared with women using neither IUD nor OC. OC users had an increased risk of CIN3+ compared with IUD users; RR 1.83 (95% CI 1.60–2.09). Results for cervical cancer pointed in the same direction but were statistically significantly increased in OC users only (Loopik *et al.*, 2020).

In summary, the estimated risks of CIN3+ associated with IUD use varied considerably depending on the comparison group included in the analysis (Table IV). An aRR of 1.51 was found when the comparison group was women not using contraceptives; an aRR close to 1 when the heterogeneous group of non-IUD users was used; and a RR of 0.55 when OC users were used. This pattern indicated that the risk of high-grade precancerous cervical lesions was higher in women requesting contraceptives than in women not requesting contraceptives or in women using OC; probably reflecting differences in sexual behavior and lifestyle. To avoid this selection bias, an internal comparison between users of various contraceptives might, therefore, be more reasonable. IUD users were consistently found to have a lower risk of CIN3+ than OC users, and our data indicated that this was true for both HIUD and CIUD users.

### Strengths and limitations

Our study used closed cohorts of women. We used national health register data, and our study is, to our knowledge, the largest investigating use of HIUD and risk of precancerous lesions. Also, this study is the first to assess the risk of progression of already present precancerous cervical lesions. Recall bias was avoided by use of register data. Linkage via unique personal identification numbers ensured complete follow-up.

A main challenge in studying the possible health consequences of contraceptive use is the choice of a control group. First, many women have used different types of contraceptives during their lifetime, most women using OC prior to other types. Second, women not registered with contraceptive use constitute a mixed and selected group, including pregnant women, sterilized women, women using barrier methods, and women with a sexual behavior different from users of contraceptives (Syrjänen *et al.*, 2006). Therefore, CIUD users and OC users were chosen as the comparison groups in our study. Consequently, the strength of our study was the comparison of three user groups.

We did not have data on sexual behavior and smoking by contraceptive user group. Therefore, we cannot exclude that some differences between user groups may have caused some residual confounding. We adjusted for age, education, and region of residence as proxies for socio-economic status. Also, we did not have data on condom use. In Denmark, condom use is less frequent than in other European countries (Nic Gabhainn *et al.*, 2009). Furthermore, as all women included in our study used contraceptives they may not see a need for additional condom use (Lauszus *et al.*, 2011; Guleria *et al.*, 2018). Consequently, condom use may be limited in our study population.

HPV-vaccinated women have a decreased risk of cervical abnormalities (Thamsborg *et al.*, 2018). We did not have individual data on HPV vaccination, but amongst the women included in our study only the youngest cohort, born in 1985, had been offered free HPV vaccination and self-paid vaccination has been rare in Denmark (Statens serum institut, 2013). Using published data on HPV vaccination coverage by birth cohort (Dillner *et al.*, 2018), we calculated that only 1.8% of women included in our study were expected to have been vaccinated against HPV. Therefore, we do not expect the lack of data on HPV vaccination to have introduced a bias.

The HIUD users seemed to adhere better to screening recommendations than the other groups, as only 16.0% in this group were excluded due to lack of baseline or follow-up diagnoses, as compared with 29.8% in the OC group, and 22.8% in the CIUD group. However, the proportions of baseline normal/abnormal diagnoses were similar in the three user groups suggesting that this missingness did not cause differences. A small number of women who have purchased an HIUD directly from a gynecologist will mistakenly have been misclassified to the CIUD group. We used OC purchase as an estimate of OC use hence, we do not know if the pills were actually consumed. However, we used only redeemed prescriptions excluding primary non-adherence, which is found to account for almost 10% of GP prescriptions (Pottegård *et al.*, 2014). If the included women did not use the OCs and thus, were not in need of contraception, we have then included women with a lower risk of cervical abnormalities, underestimating the risk of OC use.

### Clinical implications

Our findings can be used in the clinical setting when advising women in need of contraception. Women requesting contraception are at higher risk of acquiring HPV infections and of developing precancerous cervical lesions than women who do not request contraception (Lee *et al.*, 2015). For women with normal cytology at the time of insertion, we observed a 37–42% lower risk of severe precancerous cervical lesions in IUD users than in OC users that could derive from a risk associated with OC use and/or a protection associated with IUD use. In the case of protection associated with IUD use, a possible explanation could be that the IUD generates an inflammatory response in the endocervical canal, which could lower the risk of HPV infection (Castellsagué *et al.*, 2011). In agreement with other studies (Lekovich *et al.*, 2015; Averbach *et al.*, 2018), CIUD users in our study tended to have a lower risk of high-grade cervical lesions than HIUD users, which could possibly be explained by differences in their mechanism of action. CIUDs release copper ions in the uterine cavity causing the development of chronic inflammation (Ortiz and Croxatto, 2007), whereas HIUDs decrease prostaglandin levels causing suppressed local immunity, and may lead to a higher risk of persistent HPV infections (Guttinger and Critchley, 2007; Fukuyama *et al.*, 2012).

For women with high-grade precancerous cervical lesions at the time of recruitment, we found the same progression rate for HIUD users as for CIUD and OC users. For women with low-grade lesions, normal histology and abnormal cytology at recruitment, HIUD users had lower progression rates than the two other user groups. For persistence and regression of lesions at time of recruitment, no difference was observed between the three groups.

When exploring the development during the first 5 years after insertion, our findings suggested that the HIUD is an acceptable contraceptive method both for women with normal cytology at the time of insertion and for women with precancerous cervical lesions at the time of insertion. Our results did, therefore, not support the notion of the presence of cervical dysplasia as a contraindication for insertion of a HIUD. This comprehensive analysis of cervical outcomes following HIUD insertion in Danish women, thus, provided useful knowledge both for clinicians and for women preferring the HIUD, which is increasingly used for contraception and/or for treatment of heavy vaginal bleeding.

## Conclusion

In this large register-based cohort study, we found HIUD and CIUD users to have a lower risk of CIN3+ than OC users. We found little or no difference between the user groups in the risk of progression of existing precancerous cervical lesions suggesting that HIUD may also be used for women with cervical dysplasia. Our results indicated that HIUDs may safely be used for contraception or bleeding control.

## Data availability

This study used anonymized register data from the previous described Danish registers and were stored on computers at Statistics Denmark and Danish Health and Medicines Authority. Researchers have to be affiliated with a Danish authorized research environment to gain access to data. An application must be sent to the Authority describing the project and the data needed. After approval, data become available at a protected research server and transfer of data outside the server is not allowed. Researchers affiliated with/collaborating with a Danish authorized research institution are able to apply for data and reconstruct analyses made in this paper.

## Authors’ roles

M.S., E.L., J.B., and P.B. responsible for idea and data collection. All authors contributed to study design and result interpretation. G.N. analyzed data. M.S. is first author, and E.L., G.N., J.B., and P.B. contributed to manuscript preparation. All named authors approved final version.

## Funding

Financially supported by A.P. Møller Foundation for the Advancement of Medical Science (Grant no. 17-L-0236), Else and Mogens Wedell-Wedellborgs Fund (Grant no. 102-5949), Direktør Emil C. Hertz og Hustru Inger Hertz Fund (Grant no. KJR-13016) and the Fund for Development of Evidence Based Medicine in Private Specialized Practices (Grant no. A1294). Funders had no role in conduct of study.

## Conflict of interest

E.L. is principle investigator for a study with HPV-test-kits provided by Roche. M.S., G.N., P.B., and J.B. have nothing to declare.

## Supplementary Material

deab066_Supplementary_Figure_S1

deab066_Supplementary_Figure_S2

deab066_Supplementary_Table_S1

deab066_Supplementary_Table_S2

deab066_Supplementary_Table_S3

deab066_Supplementary_Data

## Supplementary data

### Computation of the length of exposure in intrauterine device groups

As each person can have several insertion codes during the study period, the cumulative exposure time was defined as the sum of all the exposure times attributable to each insertion code. As more removal codes can also appear, the exposure time attributable to each insertion code was defined as follows (here, insertion and removal codes are considered sorted in temporal order):

- If no removal code was found after an insertion code and before the subsequent insertion code/follow-up date, then the length of exposure attributable to the insertion code was defined as the time between the insertion code and the subsequent insertion code/follow-up date
- If exactly one removal code was found after an insertion code and before the subsequent insertion code/follow-up date, then the length of exposure attributable to the insertion code was defined as the time between the insertion code and the removal code
- If more than one removal code was found after an insertion code and before the subsequent insertion code/follow-up date, then the length of exposure attributable to the insertion code was defined as the time between the insertion and the last removal code
