# Supplementary material for: Risk of precancerous cervical lesions in women using a hormone-containing intrauterine device and other contraceptives: a register-based cohort study from Denmark
Source: Hum Reprod. 2021 May 11;36(7):1796–807. doi: 10.1093/humrep/deab066 (PMC8213448; doi:10.1093/humrep/deab066)
Supplement: deab066_Supplementary_Figure_S1 [file deab066_supplementary_figure_s1.pdf]

Human Reproduction, pp. 1–1, 2021
doi:10.1093/humrep/deab066

human
reproduction

SUPPLEMENTARY DATA

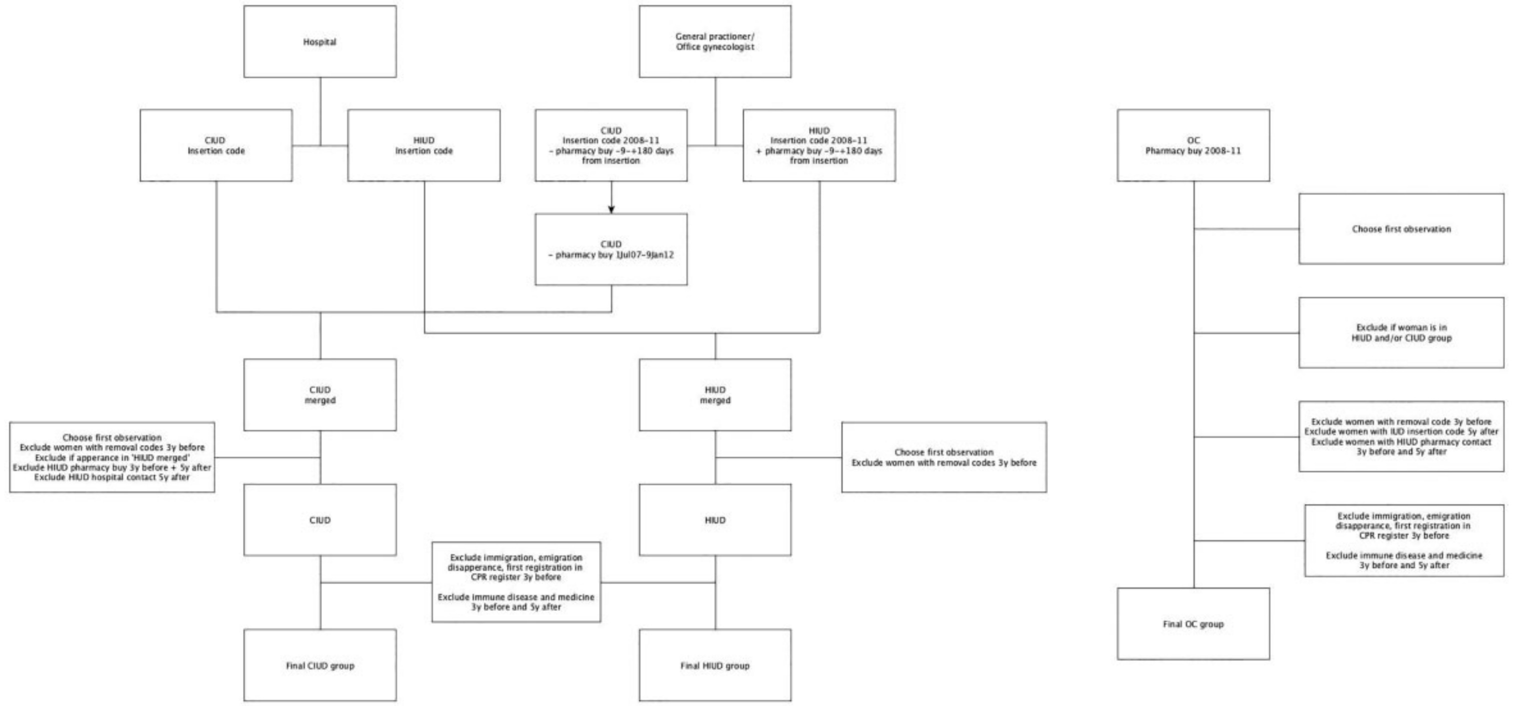

Supplementary Figure S1. Defining the three groups of women using OC, HIUD or CIUD, respectively. HIUD, hormone intrauterine device; CIUD, copper intrauterine device; OC, oral contraceptives.
