# Supplementary material for: Risk of precancerous cervical lesions in women using a hormone-containing intrauterine device and other contraceptives: a register-based cohort study from Denmark
Source: Hum Reprod. 2021 May 11;36(7):1796–807. doi: 10.1093/humrep/deab066 (PMC8213448; doi:10.1093/humrep/deab066)
Supplement: deab066_Supplementary_Figure_S2 [file deab066_supplementary_figure_s2.pdf]

**Human Reproduction, pp. 1–1, 2021**
doi:10.1093/humrep/deab066



# SUPPLEMENTARY DATA

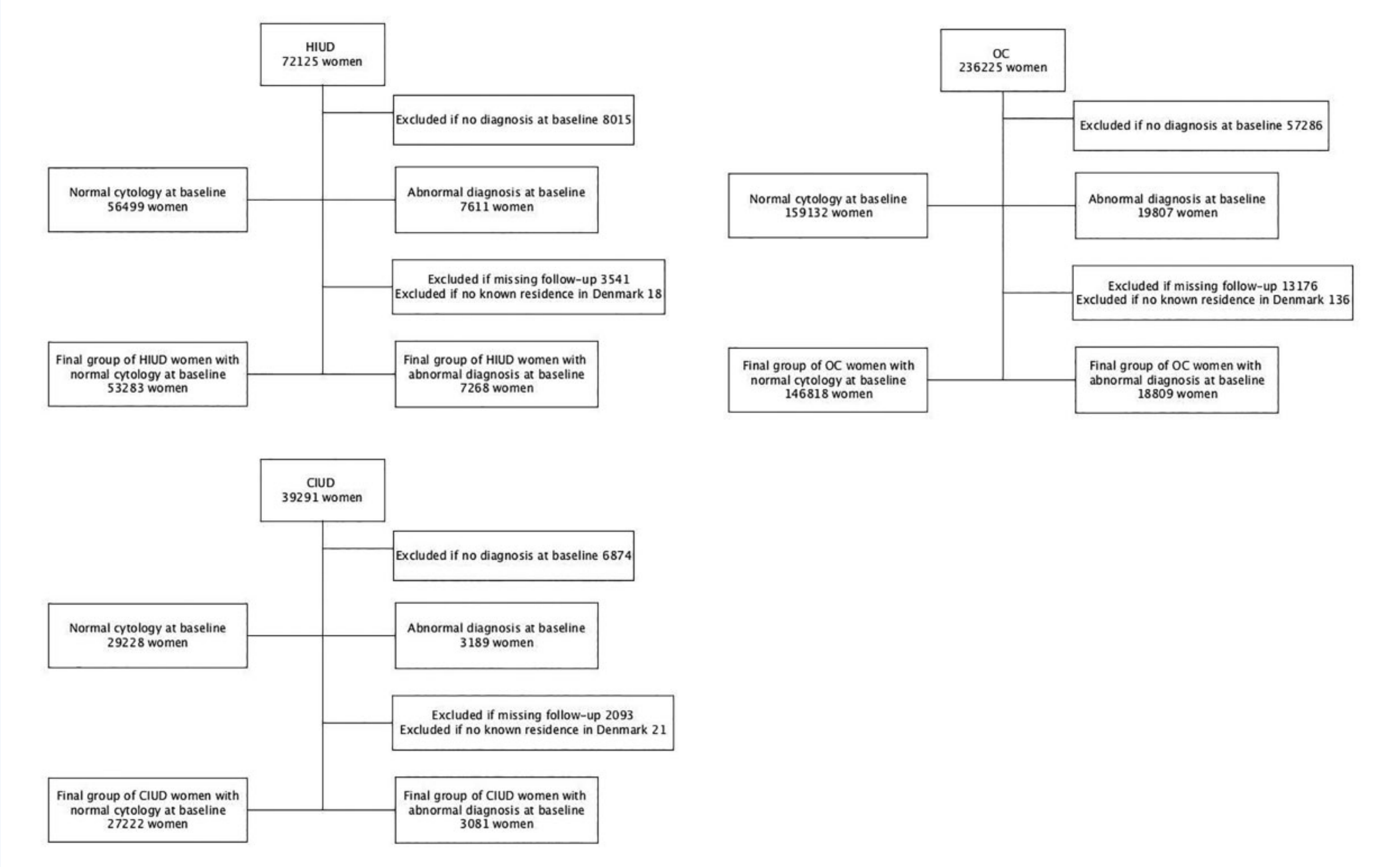


**Supplementary Figure S2.** **Division and exclusion of women into normal cytology or abnormal diagnosis at baseline in the three contraceptive groups.**
