# Supplementary material for: Risk of precancerous cervical lesions in women using a hormone-containing intrauterine device and other contraceptives: a register-based cohort study from Denmark
Source: Hum Reprod. 2021 May 11;36(7):1796–807. doi: 10.1093/humrep/deab066 (PMC8213448; doi:10.1093/humrep/deab066)
Supplement: deab066_Supplementary_Table_S1 [file deab066_supplementary_table_s1.pdf]

Human Reproduction, pp. 1–1, 2021
doi:10.1093/humrep/deab066

human reproduction

SUPPLEMENTARY DATA

Supplementary Table SI Diagnosis table with definition of progression, persistence and regression.

After<br>Before	Cancer	CIN3	CIN2	CIN1	HSIL	LSIL	ASCUS	Normal histology	Normal cytology	Total
Cancer										
CIN3										
CIN2										
CIN1										
HSIL										
LSIL										
ASCUS										
Normal histology										
Total										

Before, baseline diagnosis; After, follow-up diagnosis; CIN, cervical intraepithelial neoplasia; HSIL, high-grade squamous intraepithelial lesion; LSIL, low-grade squamous intraepithelial lesion; ASCUS, atypical squamous cells of undetermined significance.
Red = Progression.
Grey = Persistence.
Green = Regression.
