# Supplementary material for: Risk of precancerous cervical lesions in women using a hormone-containing intrauterine device and other contraceptives: a register-based cohort study from Denmark
Source: Hum Reprod. 2021 May 11;36(7):1796–807. doi: 10.1093/humrep/deab066 (PMC8213448; doi:10.1093/humrep/deab066)
Supplement: deab066_Supplementary_Table_S2 [file deab066_supplementary_table_s2.pdf]

Human Reproduction, pp. 1–1, 2021
doi:10.1093/humrep/deab066

human reproduction

SUPPLEMENTARY DATA

Supplementary Table SII **The risk of CIN3+ in HIUD, CIUD and OC users, stratified by age groups.**

Age group (years)	Groups							
	HIUD		CIUD		OC		IUDs	
	CIN3+	Total	CIN3+	Total	CIN3+	Total	CIN3+	Total
26–30	43 (2.1)	2095	53 (1.9)	2834	1739 (4.0)	43 742	96 (1.9)	4929
31–35	160 (1.9)	8630	125 (1.8)	7003	1019 (2.9)	35 517	285 (1.8)	15 633
36–40	185 (1.3)	13 746	103 (1.4)	7285	624 (2.1)	29 874	288 (1.4)	21 031
41–45	161 (1.1)	14 827	63 (1.1)	5612	305 (1.4)	21 996	224 (1.1)	20 439
46–50	82 (0.7)	11 558	24 (0.6)	3789	108 (0.8)	13 266	106 (0.7)	15 347
51–55	14 (0.6)	2427	5 (0.7)	699	15 (0.6)	2423	19 (0.6)	3126
All	645 (1.2)	53 283	373 (1.4)	27 222	3810 (2.6)	146 818	1018 (1.3)	80 505

Age group (years)	Crude relative risks (RR (95%CI))			
	HIUD versus OC	CIUD versus OC	HIUD versus CIUD	IUDs versus OC
26–30	0.52 (0.36–0.67)	0.47 (0.34–0.60)	1.10 (0.66–1.53)	0.49 (0.39–0.59)
31–35	0.65 (0.54–0.75)	0.62 (0.51–0.74)	1.04 (0.80–1.28)	0.64 (0.55–0.72)
36–40	0.64 (0.54–0.75)	0.68 (0.54–0.82)	0.95 (0.72–1.18)	0.66 (0.56–0.75)
41–45	0.78 (0.63–0.93)	0.81 (0.59–1.03)	0.97 (0.69–1.25)	0.79 (0.65–0.93)
46–50	0.87 (0.62–1.12)	0.78 (0.44–1.12)	1.12 (0.61–1.63)	0.85 (0.62–1.07)
51–55	0.93 (0.26–1.61)	1.16 (0–2.32)	0.81 (0–1.63)	0.98 (0.32–1.64)

Age group (years)	Adjusted relative risks** (aRR (95%CI))			
	HIUD versus OC	CIUD versus OC	HIUD versus CIUD	IUDs versus OC
26–30	0.57 (0.40–0.74)	0.51 (0.37–0.65)	1.12 (0.67–1.56)	0.54 (0.43–0.65)
31–35	0.61 (0.51–0.71)	0.54 (0.44–0.65)	1.12 (0.86–1.38)	0.58 (0.50–0.66)
36–40	0.61 (0.51–0.72)	0.64 (0.50–0.77)	0.96 (0.73–1.19)	0.62 (0.53–0.71)
41–45	0.70 (0.56–0.85)	0.72 (0.52–0.93)	0.97 (0.69–1.26)	0.71 (0.58–0.84)
46–50	0.70 (0.48–0.91)	0.64 (0.35–0.94)	1.08 (0.59–1.58)	0.68 (0.48–0.88)
51–55	0.74 (0.17–1.30)*	0.96 (0.01–1.90)*	0.77 (0.07–1.48)*	0.77 (0.21–1.32)*

*aRR computed using Firth logistic regression, due to small values.
**Adjusted for region of residence, education level before age 32 years, time to follow-up, and duration of IUD or OC use.
HIUD: hormone intrauterine device, CIUD: copper intrauterine device, OC: oral contraceptives.
