# Supplementary material for: Risk of precancerous cervical lesions in women using a hormone-containing intrauterine device and other contraceptives: a register-based cohort study from Denmark
Source: Hum Reprod. 2021 May 11;36(7):1796–807. doi: 10.1093/humrep/deab066 (PMC8213448; doi:10.1093/humrep/deab066)
Supplement: deab066_Supplementary_Table_S3 [file deab066_supplementary_table_s3.pdf]

Human Reproduction, pp. 1–1, 2021
doi:10.1093/humrep/deab066

human reproduction

SUPPLEMENTARY DATA

Supplementary Table SIII Diagnosis at follow-up for women with normal cytology at baseline.

Length of exposure (years)	Follow-up diagnosis	Groups: HIUD	Groups: CIUD	Groups: OC	Crude relative risks (RR (95%CI)): HIUD versus OC	Crude relative risks (RR (95%CI)): CIUD versus OC	Crude relative risks (RR (95%CI)): HIUD versus CIUD
		N = 9837	N = 5335	N = 64 746			
0-1	CIN3+	94 (1)	59 (1.1)	1135 (1.8)	0.55 (0.43–0.66)	0.63 (0.47–0.80)	0.86 (0.58–1.14)
	CIN2	61 (0.6)	20 (0.4)	488 (0.8)	0.82 (0.60–1.04)	0.50 (0.28–0.72)	1.66 (0.82–2.49)
	CIN1	72 (0.7)	61 (1.1)	572 (0.9)	0.83 (0.63–1.03)	1.29 (0.95–1.63)	0.64 (0.42–0.86)
	Normal histology	1356 (13.8)	303 (5.7)	2840 (4.4)	3.14 (2.95–3.33)	1.29 (1.15–1.44)	2.43 (2.14–2.72)
	ASCUS+	232 (2.4)	151 (2.8)	1587 (2.5)	0.96 (0.83–1.09)	1.15 (0.96–1.34)	0.83 (0.66–1.00)
	Normal cytology	7752 (78.8)	4707 (88.2)	57 642 (89)	0.89 (0.88–0.89)	0.99 (0.98–1.00)	0.89 (0.88–0.91)
	Undefined* hist	31 (0.3)	7 (0.1)	122 (0.2)	1.67 (1.01–2.33)	0.70 (0.17–1.23)	2.40 (0.43–4.37)
	Undefined* cyt	239 (2.4)	27 (0.5)	360 (0.6)	4.37 (3.66–5.08)	0.91 (0.56–1.27)	4.80 (2.90–6.71)
		**Groups**			**Crude relative risks (RR (95%CI))**		
		**HIUD N = 11 499**	**CIUD N = 6021**	**OC N = 37 571**	**HIUD versus OC**	**CIUD versus OC**	**HIUD versus CIUD**
1-2	CIN3+	128 (1.1)	70 (1.2)	1002 (2.7)	0.42 (0.34–0.49)	0.44 (0.33–0.54)	0.96 (0.68–1.23)
	CIN2	74 (0.6)	36 (0.6)	436 (1.2)	0.55 (0.42–0.69)	0.52 (0.34–0.69)	1.08 (0.65–1.50)
	CIN1	130 (1.1)	61 (1.0)	485 (1.3)	0.88 (0.71–1.04)	0.78 (0.58–0.99)	1.12 (0.78–1.45)
	Normal histology	902 (7.8)	233 (3.9)	1401 (3.7)	2.10 (1.93–2.27)	1.04 (0.90–1.18)	2.03 (1.74–2.31)
	ASCUS+	201 (1.7)	131 (2.2)	1028 (2.7)	0.64 (0.54–0.73)	0.80 (0.65–0.94)	0.80 (0.63–0.98)
	Normal cytology	9866 (85.8)	5472 (90.9)	33 011 (87.8)	0.98 (0.97–0.98)	1.03 (1.03–1.04)	0.94 (0.93–0.95)
	Undefined* hist	10 (0.1)	5 (0.1)	89 (0.2)	0.37 (0.13–0.61)	0.35 (0.03–0.67)	1.05 (0.00–2.17)
	Undefined* cyt	188 (1.6)	13 (0.2)	119 (0.3)	5.16 (3.98–6.34)	0.68 (0.29–1.07)	7.57 (3.32–11.82)
		**Groups**			**Crude relative risks (RR (95%CI))**		
		**HIUD N = 13 965**	**CIUD N = 6711**	**OC N = 24 381**	**HIUD versus OC**	**CIUD versus OC**	**HIUD versus CIUD**
2-3	CIN3+	143 (1)	81 (1.2)	677 (2.8)	0.37 (0.30–0.43)	0.43 (0.34–0.53)	0.85 (0.62–1.08)
	CIN2	88 (0.6)	49 (0.7)	299 (1.2)	0.51 (0.39–0.64)	0.60 (0.42–0.77)	0.86 (0.56–1.16)
	CIN1	139 (1.0)	53 (0.8)	312 (1.3)	0.78 (0.62–0.93)	0.62 (0.44–0.80)	1.26 (0.86–1.66)
	Normal histology	769 (5.5)	269 (4.0)	947 (3.9)	1.42 (1.29–1.55)	1.03 (0.89–1.17)	1.37 (1.19–1.57)
	ASCUS+	217 (1.6)	122 (1.8)	761 (3.1)	0.50 (0.42–0.57)	0.58 (0.47–0.69)	0.85 (0.67–1.04)
	Normal cytology	12 489 (89.4)	6101 (90.9)	21 256 (87.2)	1.03 (1.02–1.03)	1.04 (1.03–1.05)	0.98 (0.97–0.99)
	Undefined* hist	11 (0.1)	9 (0.1)	44 (0.2)	0.44 (0.15–0.72)	0.74 (0.21–1.28)	0.59 (0.07–1.10)
	Undefined* cyt	109 (0.8)	27 (0.4)	85 (0.4)	2.24 (1.61–2.87)	1.15 (0.66–1.65)	1.94 (1.12–2.76)
		**Groups**			**Crude relative risks (RR (95%CI))**		
		**HIUD N = 11 657**	**CIUD N = 6103**	**OC N = 13 137**	**HIUD versus OC**	**CIUD versus OC**	**HIUD versus CIUD**
3-4	CIN3+	147 (1.3)	93 (1.5)	535 (4.1)	0.31 (0.25–0.37)	0.37 (0.29–0.46)	0.83 (0.61–1.04)
	CIN2	101 (0.9)	43 (0.7)	205 (1.6)	0.56 (0.42–0.69)	0.45 (0.30–0.60)	1.23 (0.79–1.67)
	CIN1	160 (1.4)	65 (1.1)	212 (1.6)	0.85 (0.68–1.02)	0.66 (0.48–0.84)	1.29 (0.92–1.66)
	Normal histology	713 (6.1)	282 (4.6)	692 (5.3)	1.16 (1.04–1.28)	0.88 (0.76–1.00)	1.32 (1.14–1.50)
	ASCUS+	245 (2.1)	140 (2.3)	504 (3.8)	0.55 (0.47–0.63)	0.60 (0.49–0.71)	0.92 (0.73–1.10)
	Normal cytology	10 154 (87.1)	5448 (89.3)	10 902 (83.0)	1.05 (1.04–1.06)	1.08 (1.06–1.09)	0.98 (0.96–0.99)

(continued)

Supplementary Table SIII Continued

		Groups			Crude relative risks (RR (95%CI))		
		HIUD N = 11 657	CIUD N = 6103	OC N = 13 137	HIUD versus OC	CIUD versus OC	HIUD versus CIUD
	Undefined* hist	12 (0.1)	10 (0.2)	29 (0.2)	0.47 (0.15–0.78)	0.74 (0.21–1.28)	0.63 (0.10–1.16)
	Undefined* cyt	125 (1.1)	22 (0.4)	58 (0.4)	2.43 (1.67–3.18)	0.82 (0.42–1.22)	2.97 (1.63–4.32)

		Groups			Crude relative risks (RR (95%CI))		
		HIUD N = 6325	CIUD N = 3051	OC N = 6983	HIUD versus OC	CIUD versus OC	HIUD versus CIUD
4-5	CIN3+	133 (2.1)	70 (2.3)	461 (6.6)	0.32 (0.26–0.38)	0.35 (0.26–0.43)	0.92 (0.65–1.18)
	CIN2	89 (1.4)	49 (1.6)	201 (2.9)	0.49 (0.37–0.61)	0.56 (0.39–0.73)	0.88 (0.57–1.18)
	CIN1	140 (2.2)	56 (1.8)	213 (3.1)	0.73 (0.57–0.88)	0.60 (0.43–0.78)	1.21 (0.84–1.58)
	Normal histology	668 (10.6)	308 (10.1)	586 (8.4)	1.26 (1.13–1.39)	1.20 (1.04–1.36)	1.05 (0.91–1.18)
	ASCUS+	294 (4.6)	129 (4.2)	476 (6.8)	0.68 (0.59–0.78)	0.62 (0.50–0.74)	1.10 (0.88–1.32)
	Normal cytology	4847 (76.6)	2402 (78.7)	4982 (71.3)	1.07 (1.05–1.10)	1.10 (1.08–1.13)	0.97 (0.95–1.00)
	Undefined* hist	22 (0.4)	6 (0.2)	17 (0.2)	1.43 (0.53–2.33)	0.81 (0.06–1.56)	1.77 (0.17–3.36)
	Undefined* cyt	132 (2.1)	32 (1.0)	47 (0.7)	3.10 (2.07–4.13)	1.56 (0.86–2.25)	1.99 (1.23–2.75)

		Adjusted relative risks** (aRR (95%CI))		
Length of exposure (years)	Follow-up diagnosis	HIUD versus OC	CIUD versus OC	HIUD versus CIUD
0-1	CIN3+	0.99 (0.77–1.20)	0.88 (0.65–1.11)	1.13 (0.76–1.50)
	CIN2	1.28 (0.92–1.64)	0.62 (0.34–0.90)	2.07 (1.02–3.11)
	CIN1	1.12 (0.83–1.40)	1.57 (1.15–2.00)	0.71 (0.47–0.95)
	Normal histology	2.19 (2.04–2.34)	1.02 (0.90–1.15)	2.14 (1.87–2.41)
	ASCUS+	1.15 (0.99–1.32)	1.29 (1.08–1.51)	0.89 (0.71–1.07)
	Normal cytology	0.90 (0.89–0.91)	1.00 (0.99–1.01)	0.91 (0.89–0.92)
	Undefined* hist	1.25 (0.73–1.78)	0.58 (0.13–1.02)	2.17 (0.38–3.96)
	Undefined* cyt	2.94 (2.42–3.46)	0.84 (0.51–1.17)	3.49 (2.10–4.89)

		Adjusted relative risks** (aRR (95%CI))		
		HIUD versus OC	CIUD versus OC	HIUD versus CIUD
1-2	CIN3+	0.73 (0.59–0.87)	0.60 (0.45–0.75)	1.22 (0.87–1.58)
	CIN2	0.92 (0.68–1.16)	0.69 (0.45–0.92)	1.34 (0.80–1.87)
	CIN1	1.35 (1.07–1.64)	1.04 (0.75–1.32)	1.31 (0.91–1.71)
	Normal histology	1.99 (1.81–2.16)	1.05 (0.91–1.20)	1.89 (1.62–2.15)
	ASCUS+	0.76 (0.64–0.88)	0.91 (0.74–1.07)	0.84 (0.65–1.02)
	Normal cytology	0.95 (0.94–0.96)	1.02 (1.01–1.03)	0.94 (0.92–0.95)
	Undefined* hist	0.34 (0.11–0.56)	0.36 (0.03–0.68)	0.94 (0.00–1.95)
	Undefined* cyt	4.30 (3.23–5.37)	0.74 (0.31–1.16)	5.83 (2.54–9.12)

		Adjusted relative risks** (aRR (95%CI))		
		HIUD versus OC	CIUD versus OC	HIUD versus CIUD
2-3	CIN3+	0.67 (0.54–0.80)	0.64 (0.49–0.79)	1.05 (0.76–1.34)
	CIN2	0.83 (0.62–1.05)	0.81 (0.56–1.05)	1.04 (0.67–1.40)
	CIN1	1.19 (0.92–1.45)	0.87 (0.61–1.13)	1.36 (0.93–1.80)
	Normal histology	1.45 (1.31–1.60)	1.17 (1.01–1.33)	1.24 (1.07–1.41)
	ASCUS+	0.59 (0.49–0.68)	0.71 (0.57–0.85)	0.83 (0.65–1.01)
	Normal cytology	1.00 (0.99–1.01)	1.02 (1.01–1.03)	0.98 (0.97–0.99)

(continued)

Supplementary Table SIII Continued

		Adjusted relative risks** (aRR (95%CI)) HIUD versus OC	CIUD versus OC	HIUD versus CIUD
	Undefined* hist	0.48 (0.15–0.81)	0.82 (0.22–1.41)	0.59 (0.06–1.11)
	Undefined* cyt	2.42 (1.67–3.18)	1.44 (0.80–2.07)	1.69 (0.97–2.41)

		Adjusted relative risks** (aRR (95%CI)) HIUD versus OC	CIUD versus OC	HIUD versus CIUD
3-4	CIN3+	0.47 (0.38–0.57)	0.49 (0.38–0.61)	0.96 (0.71–1.21)
	CIN2	0.75 (0.56–0.95)	0.56 (0.37–0.75)	1.34 (0.86–1.83)
	CIN1	1.12 (0.86–1.37)	0.82 (0.59–1.06)	1.35 (0.96–1.75)
	Normal histology	1.00 (0.89–1.11)	0.81 (0.70–0.92)	1.23 (1.06–1.40)
	ASCUS+	0.57 (0.47–0.66)	0.66 (0.54–0.79)	0.86 (0.68–1.04)
	Normal cytology	1.04 (1.03–1.05)	1.07 (1.05–1.08)	0.98 (0.97–0.99)
	Undefined* hist	0.46 (0.13–0.78)	0.76 (0.20–1.32)	0.60 (0.09–1.11)
	Undefined* cyt	1.83 (1.23–2.43)	0.72 (0.36–1.07)	2.55 (1.39–3.71)

		Adjusted relative risks** (aRR (95%CI)) HIUD versus OC	CIUD versus OC	HIUD versus CIUD
4-5	CIN3+	0.52 (0.41–0.63)	0.47 (0.35–0.59)	1.10 (0.78–1.42)
	CIN2	0.74 (0.54–0.94)	0.71 (0.49–0.94)	1.04 (0.67–1.40)
	CIN1	1.00 (0.77–1.24)	0.76 (0.53–0.99)	1.32 (0.91–1.73)
	Normal histology	1.10 (0.98–1.22)	1.16 (1.01–1.32)	0.94 (0.82–1.07)
	ASCUS+	0.75 (0.63–0.86)	0.73 (0.59–0.87)	1.03 (0.81–1.24)
	Normal cytology	1.05 (1.02–1.07)	1.08 (1.05–1.11)	0.97 (0.94–1.00)
	Undefined* hist	1.11 (0.36–1.86)	0.74 (0.04–1.44)	1.49 (0.13–2.85)
	Undefined* cyt	2.98 (1.92–4.03)	1.53 (0.84–2.22)	1.95 (1.19–2.70)

Data are crude and adjusted RR with 95% CI. Stratified by length of exposure.
*Undefined = Other/unsatisfactory.
**Adjusted for age, region of residence, education level before age 32 years, and time to follow-up.
