# Supplementary material for: Risk of precancerous cervical lesions in women using a hormone-containing intrauterine device and other contraceptives: a register-based cohort study from Denmark
Source: Hum Reprod. 2021 May 11;36(7):1796–807. doi: 10.1093/humrep/deab066 (PMC8213448; doi:10.1093/humrep/deab066)
Supplement: deab066_Supplementary_Data [file deab066_supplementary_data.pdf]

Human Reproduction, pp. 1–1, 2021
doi:10.1093/humrep/deab066

human reproduction
SUPPLEMENTARY DATA

Supplementary data

Computation of the length of exposure in intrauterine device groups

As each person can have several insertion codes during the study period, the cumulative exposure time was defined as the sum of all the exposure times attributable to each insertion code. As more removal codes can also appear, the exposure time attributable to each insertion code was defined as follows (here, insertion and removal codes are considered sorted in temporal order):

- If no removal code was found after an insertion code and before the subsequent insertion code/follow-up date, then the length of exposure attributable to the insertion code was defined as the time between the insertion code and the subsequent insertion code/follow-up date
- If exactly one removal code was found after an insertion code and before the subsequent insertion code/follow-up date, then the length of exposure attributable to the insertion code was defined as the time between the insertion code and the removal code
- If more than one removal code was found after an insertion code and before the subsequent insertion code/follow-up date, then the length of exposure attributable to the insertion code was defined as the time between the insertion and the last removal code
